# Vertebrobasilar Junction Angle Over 90°: A Potential Imaging Marker Associated With Vertebrobasilar Atherosclerosis

**DOI:** 10.3389/fnins.2021.789852

**Published:** 2022-01-05

**Authors:** Jia Li, Wen-Jie Yang, Lu Zheng, Heng Du, Winnie Chiu-Wing Chu, Thomas Wai-Hong Leung, Xiang-Yan Chen

**Affiliations:** ^1^Department of Critical Care Medicine, Ruijin Hospital, Shanghai Jiao Tong University School of Medicine, Shanghai, China; ^2^Department of Health Technology and Informatics, The Hong Kong Polytechnic University, Kowloon, Hong Kong SAR, China; ^3^Department of Diagnostic Radiology and Nuclear Medicine, University of Maryland, Baltimore, Baltimore, MD, United States; ^4^Department of Neurology, The Third Affiliated Hospital of Sun Yat-sen University, Guangzhou, China; ^5^Department of Imaging and Interventional Radiology, Prince of Wales Hospital, The Chinese University of Hong Kong, Shatin, Hong Kong SAR, China; ^6^Division of Neurology, Department of Medicine and Therapeutics, Prince of Wales Hospital, The Chinese University of Hong Kong, Shatin, Hong Kong SAR, China

**Keywords:** intracranial atherosclerosis, vertebrobasilar circulation, vertebrobasilar junction angle, cerebral vascular variation, plaque imaging feature

## Abstract

**Objective:** Whether the cerebral vascular variations play an important role in the progression of intracranial atherosclerosis is yet largely unclear. We aimed to investigate the relationship between the magnitude of the vertebrobasilar junction (VBJ) angle and the imaging features of vertebrobasilar artery atherosclerosis.

**Methods:** Adult patients with acute ischemic stroke or transient ischemic attack undergoing a 3.0-tesla vessel wall magnetic resonance imaging (VW-MRI) scanning were consecutively included. Imaging features of vertebrobasilar artery atherosclerosis were assessed on the reconstructed short axis of VW-MRI at the most stenotic site. The VBJ angle degree was measured on magnetic resonance angiography and classified into the angle ≥90° or <90°.

**Results:** Among 68 patients (mean age = 63.5 ± 9.4 years old; 63.2% were male) with vertebrobasilar atherosclerosis, 33 had a VBJ angle ≥90° and 35 had a VBJ angle <90°. Compared to the vertebrobasilar plaques with VBJ angle <90°, those with VBJ angle ≥90° had a heavier plaque burden (84.35 vs. 70.58%, *p* < 0.001) and higher prevalence of intraplaque hemorrhage (17.1 vs. 3.3%, *p* = 0.01). In the regression analyses, the VBJ angle ≥90° was also robustly associated with plaque burden (odds ratio, 1.11; 95% confidential interval, 1.043–1.18; *p* = 0.001) and intraplaque hemorrhage (odds ratio, 5.776; 95% confidential interval, 1.095–30.46; *p* = 0.039) of vertebrobasilar atherosclerosis.

**Conclusion:** The VBJ angle over 90° might aggravate the vessel wall condition of the atherosclerotic vertebrobasilar arteries, which might serve as a potential risk factor for vertebrobasilar atherosclerosis.

## Introduction

Ischemic stroke (IS) due to intracranial large artery atherosclerosis poses a major threat to the global public health and economy (Kaul et al., 2018; Kim et al., 2020; Wang et al., 2020). Up to 25% of acute ischemic events entail the brain tissues supported by the vertebrobasilar artery system and still carries increasing risks of stroke-related disability and stroke recurrence (Savitz and Caplan, 2005; Sparaco et al., 2019).

Geometric variants commonly exist in the vertebrobasilar artery system and may exert a strong influence on the formation of vertebrobasilar atherosclerosis mainly *via* the direct impact on the vertebrobasilar circulation hemodynamics (Yu et al., 2018; Xu et al., 2019; Zhou et al., 2020). Notably, the confluence angles of the vertebrobasilar junction (VBJ) vary from 10° to 160° (Ravensbergen et al., 1996, 1998). Previous research showed that patients with deep pontine lacunar infarction had significantly larger VBJ angles than healthy individuals (Jeong et al., 2015). Furthermore, the larger VBJ angles were also revealed in robust relevance to the hemodynamic alteration, thereby leading to the proneness to vertebrobasilar atherosclerosis in the numerical and experimental models (Ravensbergen et al., 1996, 1998; Zhang et al., 2016). Nevertheless, little clinical evidence from stroke patients could be provided to ascertain the relationship between the varying VBJ angle degrees and the progression of vertebrobasilar atherosclerosis.

With the utilization of high-resolution vessel wall magnetic resonance imaging (VW-MRI), the imaging features of vertebrobasilar artery atherosclerosis can be evaluated quantitatively and qualitatively in patients with acute ischemic stroke (AIS) attributed to intracranial large-artery atherosclerotic stenosis. In this hospital-based study, we aimed to determine the association of the VBJ angle magnitude with the imaging characterization of vertebrobasilar atherosclerosis assessed on VW-MRI.

## Materials and Methods

### Study Population

Consecutive adult patients with IS or transient ischemic attack (TIA) who underwent VW-MRI at the Prince of Wales Hospital from 2015 to 2020 for intracranial large artery stenosis were retrospectively included in this study. The inclusion criteria for this study were as follows: (1) First-ever AIS or TIA within 7 days; (2) Presence of the bilateral vertebral arteries (VAs) and the basilar artery (BA) shown on magnetic resonance angiography (MRA); (3) The intracranial segments of the VAs completely covered by VW-MRI; (4) Atherosclerotic plaques on the intracranial VA and the BA detected by VW-MRI; (5) Good image quality for the imaging analyses. Subjects were excluded when meeting the following conditions: (1) Non-atherosclerotic stenosis, such as moyamoya disease, vasculitis, and dissection; (2) Clinical evidence of cardioembolism, including valvular heart disease and atrial fibrillation; (3) Coexistent moderate to severe carotid artery stenosis; (4) History of vascular malformation, brain tumor, or cerebral interventional or vascular surgical procedure. The patient baseline clinical data were recorded (age, sex, hypertension, hyperlipidemia, diabetes, and current smoking status). The study was approved by the Joint Chinese University of Hong Kong-New Territories East Cluster Clinical Research Ethics Committee (the Joint CUHK-NTEC CREC, No. 2015.011) and followed the 1975 Declaration of Helsinki. The written informed consents were signed by all the patients or their family members.

### Imaging Protocol

All VW-MRI exams were performed using a 3.0-tesla Achieva MR system (Philips Healthcare, Cleveland, OH, United States) with an 8-channel head coil in this study. As described in our previous VW-MRI study (Li et al., 2020), the imaging protocol included a 3-dimensional T1-weighted (T1w) Volumetric ISotropically Turbo spin echo Acquisition (VISTA) sequence before and after the administration of a gadolinium-containing contrast agent (Dotarem, Gadoteric acid 0.5 mmol/ml; Guerbet, Roissy CdG Cedex, France) (0.1 ml/kg to each subject) and a 3-dimensional Time-Of-Flight (TOF) MRA sequence. The T1w VISTA sequence parameters were as follows: field of view (FOV) 200 mm × 167 mm × 45 mm, acquired resolution 0.6 mm × 0.6 mm × 1.0 mm, and repetition time (TR)/echo time (TE) 1,500/36 ms. The TOF MRA sequence was acquired using FOV 200 mm × 200 mm × 56 mm, acquired resolution 0.4 mm × 0.6 mm × 0.7 mm, and TR/TE 23/3.5 ms.

### Measuring the Vertebrobasilar Junction Angle Magnitude

**FIGURE 1:**
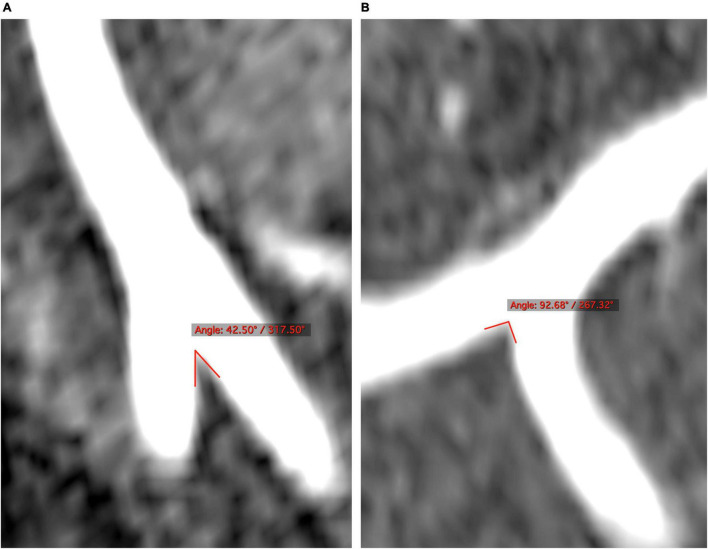
The degrees of the VBJ confluence angles are examined on the 3-dimensional reconstructed cross sections of MRA. **(A,B)** The VBJ angles less than and more than 90°, respectively. MRA, magnetic resonance angiography; VBJ, vertebrobasilar junction.

Two raters (JL and HD) blind to the patient clinical information independently measured the degrees of the VBJ angles on TOF MRA imaging using OsiriX DICOM Viewer (Geneva, Switzerland) (Figure 1). The VBJ angle referred to the relevant angle between the two inner vessel walls of the bilateral VAs (Ravensbergen et al., 1998). The measurement of the VBJ angle magnitude was carried out independently of the subsequent assessment of the vertebrobasilar artery plaques.

### Quantitatively and Qualitatively Evaluating the Vertebrobasilar Plaques

**FIGURE 2:**
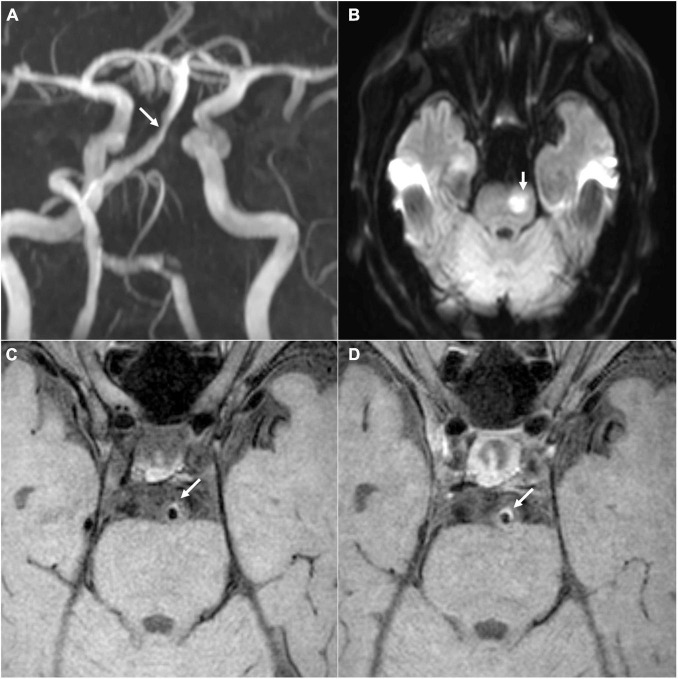
A representative case of acute ischemia in vertebrobasilar circulation. **(A)** The MRA image shows stenosis in the BA (white arrow). **(B)** The diffusion-weighted image shows acute infarction in the BA territory (white arrow). **(C,D)** The pre- and post-contrast T1-weighted images show the BA plaque as a symptomatic lesion, respectively (white arrows). BA, basilar artery; MRA, magnetic resonance angiography.

Another two raters (W-JY and LZ) also blind to the patient clinical data independently determined the imaging features of vertebrobasilar atherosclerosis on VW-MRI (Figure 2). Atherosclerotic plaques along the bilateral intracranial VA segments and the BA were identified as vessel wall thickening on the matched pre- and post-contrast T1w images, according to a previously published definition (Yang et al., 2021).

The quantitative measurements of vertebrobasilar artery plaques were performed on the reconstructed cross-sectional images at the narrowest luminal site by using VesselMass software (Leiden University Medical Center, Netherlands) based on the prior methods (Guo et al., 2018; Wang et al., 2018; Yang et al., 2020). The adjacent plaque-free, non-tortuous, normal vessel segment proximal or distal to the lesion was used as the reference site. The vessel–cerebrospinal fluid interface was manually traced to measure vessel area (VA_*measure*_). The blood–intima interface was used for determining lumen area (LA). Wall area (WA) was defined as VA_*measure*_ – LA. Luminal stenosis percentage was calculated as (1 – lesion LA/reference LA) × 100%. Plaque burden was defined as (lesion WA/lesion VA_*measure*_) × 100%. Wall area index was determined by lesion WA/reference WA. Remodeling index (RI) was defined as lesion VA_*measure*_/reference VA_*measure*_. The remodeling pattern was designated as positive (RI ≥ 1.05) or non-positive (RI < 1.05). Plaque eccentricity index was defined as (maximum wall thickness – minimum wall thickness)/maximum wall thickness. Each plaque was classified as eccentric (eccentricity index ≥0.5) or concentric (eccentricity index <0.5).

A round area of 10–12 mm^2^ at the normal gray matter near the plaque lesion was manually drawn on both pre- and post-contrast T1w images to measure the signal intensity of gray matter (SI _*gray*–matter_). The signal intensity of plaque (SI _*plaque*_) normalized to SI _*gray*–matter_ was measured on the matched pre- and post-contrast T1w images to determine the plaque contrast enhancement: Plaque enhancement index = [(SI _*plaque*_/SI _*gray*–matter_ on post-contrast T1w images) – (SI _*plaque*_/SI _*gray*–matter_ on pre-contrast T1w images)]/(SI _*plaque*_/SI _*gray*–matter_ on pre-contrast T1w images) × 100%.

Hypointensity or hyperintensity in the plaque by comparison with the signal intensity of the adjacent normal gray matter tissue on the pre-contrast T1w image was recorded (Yang et al., 2016). The presence of intraplaque hemorrhage (IPH) was recognized as a region of high signal within the plaque (over 150% of the signal intensity of adjacent vessel-wall area) on the pre-contrast T1w images (Yang et al., 2020).

A plaque was classified as symptomatic if it was the only or the most enhanced plaque in the territory of brain infarct or neurological symptom (Yang et al., 2020). It was defined as asymptomatic plaque if the plaque was out of the territory of brain infarct or neurological symptom or the plaque was not the most enhanced in the corresponding ischemic territory (Yang et al., 2020).

### Statistical Analysis

All statistical data were analyzed using the SPSS version 26.0 (IBM, NY, United States). The distribution of the VBJ angles was displayed using GraphPad Prism 8.0 (GraphPad Software Inc., San Diego, CA, United States). Variables were shown as mean ± standard deviation (SD), median [interquartile range (IQR)], or number (percentage), when appropriate. The baseline clinical features of subjects with vertebrobasilar artery atherosclerosis were compared between the VBJ angles ≥90° and the angles <90° by using *t*-test, chi-square test, or Fisher’s exact test. The imaging characterizations of vertebrobasilar atherosclerotic plaques were compared between the cases with VBJ angle ≥90° and those with VBJ angle <90° by using Mann–Whitney *U* test, chi-square test, or Fisher’s exact test. Univariate and multivariate logistic regression analyses were carried out to further estimate the relationship between the VBJ angle magnitude and the imaging features of vertebrobasilar artery atherosclerosis. A *p*-value < 0.05 was regarded as statistically significant. Inter-rater reliability was determined by intraclass correlation or Cohen κ coefficient with 95% confidence interval (CI). A coefficient >0.81 was considered as excellent.

## Results

### Patient Demographic and Clinical Characteristics

A total of 68 patients (mean age, 63.53 ± 9.42 years; 43 male) with vertebrobasilar artery atherosclerosis were included in this study. The patient clinical characteristics on admission were summarized in Table 1.

**TABLE 1:** Baseline clinical characteristics of patients with vertebrobasilar artery atherosclerosis between the VBJ angles ≥90° and <90°.

Parameters	All subjects (*n* = 68)	Subjects with VBJ angle ≥90° (*n* = 33)	Subjects with VBJ angle <90° (*n* = 35)	*p*-value
Age, years, mean ± SD	63.53 ± 9.42	62.97 ± 9.69	64.06 ± 9.25	0.638
Male/female, n	43/25	22/11	21/14	0.569
Hypertension, n (%)	54 (79.4%)	26 (78.8%)	28 (80.0%)	0.902
Hyperlipidemia, n (%)	39 (57.4%)	21 (63.6%)	18 (51.4%)	0.309
Diabetes, n (%)	27 (39.7%)	11 (33.3%)	16 (45.7%)	0.297
Smoking, n (%)	18 (26.5%)	9 (27.3%)	9 (25.7%)	0.884
Index event				0.107
Stroke, n (%)	61 (89.7%)	32 (97.0%)	29 (82.9%)	
TIA, n (%)	7 (10.3%)	1 (3.0%)	6 (17.1%)	

*SD, standard deviation; TIA, transient ischemic attack; VBJ angle, vertebrobasilar junction angle.*

**FIGURE 3:**
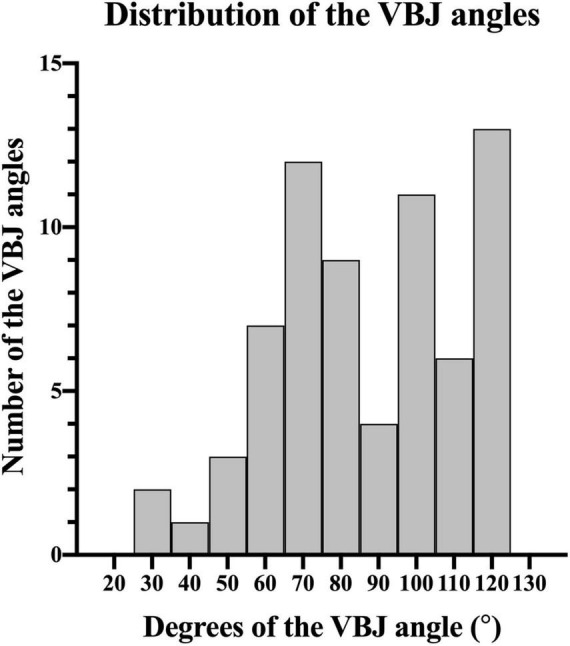
Histograms showing the distribution of the VBJ angles in patients with vertebrobasilar artery atherosclerosis. VBJ, vertebrobasilar junction.

The degrees of the VBJ angles varied from 29.45° to 124.20° (median, 85.18°; IQR, 68.34°–108.02°) in patients with vertebrobasilar atherosclerosis (Figure 3). According to the VBJ angle magnitude exceeding 90° or not, all the patients were subsequently categorized into two groups: 33 with VBJ angle ≥90° and 35 with VBJ angle <90°. Yet, no statistical significance was observed in the difference of the baseline clinical features between the two groups (all *p*-values > 0.05), as shown in Table 1.

### Vertebrobasilar Artery Atherosclerosis and the Vertebrobasilar Junction Angle Magnitude

In total, 131 vertebrobasilar atherosclerotic plaques were detected in all the subjects, including 70 within the VBJ angles ≥90° and 61 within the VBJ angles <90°. The comparisons of the vertebrobasilar plaque imaging features between the groups with VBJ angle ≥90° and <90° were illustrated in Table 2.

**TABLE 2:** Imaging characteristics of vertebrobasilar artery atherosclerosis between the VBJ angles ≥90° and <90°.

Features	Plaques with VBJ angle ≥90° (*n* = 70)	Plaques with VBJ angle <90° (*n* = 61)	*p*-value
Symptomatic status, n (%)	14 (20.0%)	3 (4.9%)	0.010
Hypointensity signal, n (%)	36 (51.4%)	19 (31.1%)	**0.019**
Hyperintensity signal, n (%)	19 (27.1%)	14 (23.0%)	0.581
IPH, n (%)	12 (17.1%)	2 (3.3%)	**0.010**
Plaque wall morphology			0.272
Eccentric, n (%)	40 (57.1%)	29 (47.5%)	
Concentric, n (%)	30 (42.9%)	32 (52.5%)	
Arterial remodeling			0.740
Positive, n (%)	51 (72.9%)	46 (75.4%)	
Non-positive, n (%)	19 (27.1%)	15 (24.6%)	
Luminal stenosis, %, median (IQR)	73.89 (53.81-85.12)	45.68 (35.24-66.32)	**<0.001**
Plaque burden, %, median (IQR)	84.35 (75.24-89.88)	70.58 (64.89-77.73)	**<0.001**
Wall area index, median (IQR)	35.89 (12.43-63.88)	31.80 (12.67-47.45)	0.190
Remodeling index, median (IQR)	1.31 (1.03-1.98)	1.45 (1.03-1.73)	0.761
Eccentricity index, median (IQR)	0.57 (0.36-0.69)	0.48 (0.33-0.66)	0.299
Enhancement index, %, median (IQR)	38.83 (18.42-57.91)	32.43 (12.37-45.34)	0.131

*IPH, intraplaque hemorrhage; IQR, interquartile range; VBJ angle, vertebrobasilar junction angle.*

*The bold values are highlighted that they are less than 0.05.*

As shown in Table 2, the VBJ angles ≥90° possessed more vertebrobasilar artery plaques with symptomatic status (20.0 vs. 4.9%, *p*-value = 0.01), hypointensity signal (51.4 vs. 31.1%, *p*-value = 0.019), and IPH (17.1 vs. 3.3%, *p*-value = 0.01) than the VBJ angles <90°. However, no statistical significance was found in the difference of plaque hyperintensity signal, plaque wall morphology, or arterial remodeling pattern between the VBJ angles ≥90° and the angles <90° (all *p*-values > 0.05; Table 2).

Moreover, compared to the vertebrobasilar plaques with VBJ angle <90°, those with VBJ angle ≥90° had significantly higher percentages of luminal stenosis (73.89 vs. 45.68%, *p*-value < 0.001; Table 2) and plaque burden (84.35 vs. 70.58%, *p*-value < 0.001; Table 2). There were no significant differences observed in other plaque imaging characteristics between the two groups, such as wall area index, RI, plaque eccentricity index, and plaque enhancement index (all *p*-values > 0.05; Table 2).

### Univariate and Multivariate Analyses

In the univariate analysis, the VBJ angles ≥90° were related to plaque hypointensity signal (odds ratio, 2.341; 95% CI, 1.143–4.792; *p*-value = 0.02), IPH (odds ratio, 6.103; 95% CI, 1.308–28.476; *p*-value = 0.021), luminal stenosis (odds ratio, 1.048; 95% CI, 1.028–1.07; *p*-value < 0.001), and plaque burden (odds ratio, 1.137; 95% CI, 1.085–1.191; *p*-value < 0.001) in vertebrobasilar artery atherosclerosis, as displayed in Table 3.

**TABLE 3:** Univariate and multivariate regression for independent correlation between the imaging characteristics of vertebrobasilar atherosclerosis and the VBJ angles ≥90°.

	Univariate model		Multivariate model*^a^*	
	OR 95% CI	*p*-value	OR 95% CI	*p*-value
Hypointensity signal	2.341 (1.143–4.792)	**0.02**	1.395 (0.569–3.418)	0.466
IPH	6.103 (1.308–28.476)	**0.021**	5.776 (1.095–30.46)	**0.039**
Luminal stenosis	1.048 (1.028–1.07)	**<0.001**	1.015 (0.986–1.044)	0.319
Plaque burden	1.137 (1.085–1.191)	**<0.001**	1.11 (1.043–1.18)	**0.001**

*CI, confidential interval; IPH, intraplaque hemorrhage; OR, odds ratio; VBJ angle, vertebrobasilar junction angle.*

*^a^Adjusted for age, sex, hypointensity signal, intraplaque hemorrhage, luminal stenosis, and plaque burden.*

*The bold values are highlighted that they are less than 0.05.*

In the multivariate analysis adjusted for potential confounders, the VBJ angles ≥90° were still shown in strong association with IPH (odds ratio, 5.776; 95% CI, 1.095–30.46; *p*-value = 0.039; Table 3) and plaque burden (odds ratio, 1.11; 95% CI, 1.043–1.18; *p*-value = 0.001; Table 3) caused by vertebrobasilar atherosclerosis. Yet, the VBJ angles ≥90° were not strongly associated with plaque hypointensity signal and luminal stenosis in vertebrobasilar atherosclerosis.

### Inter-Rater Reliability

Inter-rater reliability on the measurement of the VBJ angle magnitude was excellent (the intra-observer reliability: coefficient = 0.907, 95% CI 0.814–0.955; the inter-observer reliability: coefficient = 0.860, 95% CI 0.728–0.931). Inter-rater reliability on the quantitative and qualitative evaluation of the plaque imaging features was substantial to excellent, which was reported in our previous VW-MRI studies (Dieleman et al., 2016; Yang et al., 2016).

## Discussion

In this hospital-based study, firstly, we found that the plaques in the VBJ angles over 90° were more likely to cause acute infarcts and/or acute stroke symptoms in vertebrobasilar circulation. Secondly, the VBJ angles above 90° were observed in significant correlation with higher prevalence of plaque hypointensity signal and IPH and higher degrees of luminal stenosis and plaque burden in vertebrobasilar artery atherosclerosis. Thirdly, logistic regression analyses further revealed that the relevance of the VBJ angles more than 90° to IPH and plaque burden in vertebrobasilar atherosclerosis remained robust.

Using 3-dimensional TOF MRA imaging, our study showed the striking variations in the magnitude of the VBJ angles among patients with vertebrobasilar artery atherosclerosis, ranging from 29.45° to 124.20°. This observation was consistent with the previous research reporting that the geometric variability of the VBJ angle magnitude was generally found in autopsy human brains (Ravensbergen et al., 1996, 1998). Interestingly, a larger VBJ angle magnitude was subsequently highlighted to have an effect on the development of atherosclerotic plaques in the experimental and numerical analyzing models (Ravensbergen et al., 1996, 1998; Zhang et al., 2016).

Two causes could be given. First, the degrees of the VBJ angle might strongly affect the patterns of cerebral hemodynamics that acted on the intracranial vessel walls (Ravensbergen et al., 1996, 1998; Ritter and Ringelstein, 2002). Notably, the complex patterns of blood flow and the reduced levels of wall shear stress were revealed in the models of the larger VBJ angles, which led to the formation of atherosclerosis (Ravensbergen et al., 1996, 1998). Second, the VBJ angle magnitude might also make a direct impact on the activity of vascular smooth muscle cells (VSMCs) of the vertebrobasilar arteries to develop atherosclerosis (Zhang et al., 2016). In particular, the VBJ angle structure of 90°could significantly attenuate the reaction of VSMCs to the local change in the hemodynamic force and result in the higher expressions of the pro-atherosclerotic mediators in the VSMCs (Zhang et al., 2016). Accordingly, we speculated that the geometry of the VBJ angles over 90° might deteriorate the vessel wall conditions of the vertebrobasilar arteries to grow atherosclerotic plaques mainly through the influence on the hemodynamic pattern and the vascular activity.

This hypothesis could be largely supported by our findings. Firstly, the VBJ angles exceeding 90° were robustly related to heavier plaque burden of the atherosclerotic vertebrobasilar arteries. It is notable that the degree of plaque burden in intracranial large artery atherosclerosis was suggested as a better indicator of the luminal narrowing severity than other conventional imaging values (Wang et al., 2019; Ran et al., 2020; Wu et al., 2020). Secondly, a significant increase in the prevalence of the vertebrobasilar plaques with IPH was found in the VBJ angles over 90°. As a representative imaging marker for high-risk plaque, IPH in vertebrobasilar circulation atherosclerosis was revealed in significant correlation with the plaque vulnerability and rupture, regardless of whether the stenotic grade was high or low (Shi et al., 2018, 2020; Zhu et al., 2018). More importantly, the logistic regression models in our study indicated that the VBJ angles above 90° were independently related to plaque burden and IPH in vertebrobasilar atherosclerosis. Therefore, the VBJ angle structure over 90° was robustly associated with a high-risk vessel wall condition of vertebrobasilar artery atherosclerosis.

Our observations are of potential clinical importance. Although further verification is required, we initially find that the symptomatic atherosclerotic plaques causing acute infarctions and/or acute ischemic signs in vertebrobasilar circulation were more likely to be located in the VBJ angles above 90°, rather than those below 90°. Given the stroke mechanisms varying among symptomatic patients with vertebrobasilar artery atherosclerotic stenosis (Samaniego et al., 2019; Schaafsma et al., 2019), subsequent research is urgently needed to determine if the VBJ angle geometry exceeding 90° is an independent risk factor for stroke occurrence and stroke patterns.

The present study had limitations. Firstly, any causal relationship between the VBJ angle magnitude and vertebrobasilar artery atherosclerosis could not be concluded from this observational study. Secondly, despite the consistency with the prior findings of a low prevalence of symptomatic vertebrobasilar artery atherosclerotic disease (Marquardt et al., 2009; Kim et al., 2013), further exploration of the association between the VBJ angle degrees and the infarct patterns was not allowed in this study due to a relatively small study population. Thirdly, we did not perform any flow dynamic analysis but may inspire future study directions.

## Conclusion

Utilizing high-resolution VW-MRI, significant differences in the imaging characteristics of vertebrobasilar artery atherosclerosis were observed between the VBJ angles more than 90° and the angles less than 90°. The structure of the larger degrees of the VBJ angle might be a risk factor for vertebrobasilar atherosclerosis. Further research is still required to investigate the underlying hemodynamic mechanisms depending on the increasing magnitude of the VBJ angles.

## Data Availability Statement

The raw data supporting the conclusions of this article will be made available by the authors, without undue reservation.

## Ethics Statement

The studies involving human participants were reviewed and approved by the Joint Chinese University of Hong Kong-New Territories East Cluster Clinical Research Ethics Committee. The patients/participants provided their written informed consent to participate in this study.

## Author Contributions

JL analyzed imaging data and drafted the manuscript. W-JY and LZ recruited patients and analyzed imaging data. HD participated in analyzing imaging data. WC and TL participated in study coordination and patient recruitment. X-YC conceived the study, participated in its design and coordination, and revised the manuscript. All authors contributed to the article and approved the submitted version.

## Conflict of Interest

The authors declare that the research was conducted in the absence of any commercial or financial relationships that could be construed as a potential conflict of interest.

## Publisher’s Note

All claims expressed in this article are solely those of the authors and do not necessarily represent those of their affiliated organizations, or those of the publisher, the editors and the reviewers. Any product that may be evaluated in this article, or claim that may be made by its manufacturer, is not guaranteed or endorsed by the publisher.
